# Mycosynthesis of silver nanoparticles by *Aspergillus templicola* OR480102: a multifaceted approach for antibacterial, anticancer, and scratch assay applications

**DOI:** 10.1186/s12896-025-00982-6

**Published:** 2025-06-11

**Authors:** Marwa M. Abdel-Kareem, Maysa M. A. Ali, Abd El-Latif Hesham, Hossam E. F. Abdel-Raheam, Marwa Obiedallah

**Affiliations:** 1 Botany & Microbiology Department, Faculty of Science, Sohag University, Sohag, Egypt; 2 Botany & Microbiology Department, Faculty of Science, Assuit University, Assuit, Egypt; 3 Department of Genetics, Faculty of Agriculture, Beni-Suef University, Beni-Suef, 62521 Egypt; 4 Department of Food Sciences, Faculty of Agriculture, Beni-Suef University, Beni-Suef, 62521 Egypt

**Keywords:** Silver nanoparticles, *Aspergillus templicola*, Characterization, Antibacterial, Apoptosis, Scratch, Anticancer

## Abstract

**Background:**

Regarding their distinct physico-chemical and bioactivity characteristics, silver nanoparticles ‘AgNPs’ are extensively utilized in numerous scientific purposes.

**Results:**

Within this current investigation, for the first time, we evaluated how the extracellular extract of the isolate MAK223 generated exceptionally fixed AgNPs. The isolate was genetically identified as *Aspergillus templicola* OR480102. The generated AgNPs’ physico-chemical characteristics were assessed using ultraviolet-vis spectroscopy, transmission electron microscopy (TEM), and Fourier transform infrared spectrometry (FT-IR). The maximum absorption in the UV-vis spectrum was obtained at 420 nm, matching the silver nanoparticles’ surface plasmon absorbance. *A. templicola* OR480102 produced uniformly dispersed AgNPs between 5 and 25 nm with a mean dimension of 17.78537 ± 1.36 nm using TEM. FT-IR analysis identified functional groups (e.g., -OH, C = O) in the fungal filtrate that mediate AgNP synthesis and capping. To verify AgNPs stability, the dynamic light scattering (DLS) approach is employed. Optimal conditions for AgNPs synthesis were 10 days of incubation, one mM silver nitrate concentration, pH 11, and elevated temperatures. AgNPs demonstrated efficacy against clinically relevant pathogens: *S*. *typhimurium* ‘ATCC 14028’, *B*. *subtilis* ‘ATCC 6633’, *S*. *aureus* ‘ATCC 25923’, and *E*. *coli* ‘ATCC 29213’ were used in the study. Also, using AgNPs derived from the filtrate of *A. templicola* OR480102 shows significant potential as a novel therapeutic approach against breast cancer cells ‘MCF-7’. The scratch assay of ‘MCF-7’ cells demonstrates the suppressive impact of AgNPs for these cell lines during proliferation by promoting apoptosis and reducing cell migration.

**Conclusion:**

Based on physico-chemical characteristics of AgNPs’ and their antimicrobial and anticancer activities, it cleared that the selected strain *Aspergillus templicola* OR480102 is a promising producer of stable AgNPs’ with significant bioactivities which could be applicable in different fields.

## Introduction

The future of nanoscience is promising as it is a thriving field. It deals with materials in dimensions ranging from 1 to 100 nm, whose merits differ from those of their more substantial equivalents [1]. Unique chemical, physical, electrical, and magnetic properties are displayed in nanoscale-sized materials, offering them a formidable potential for use in various agricultural applications [2], medicine [3], and several significant technology fields [4].

Nanomaterials can be created through physical, chemical, and biological approaches.

Sever conditions, encompassing a high concentration of toxic pollutants, elevated pressures, and temperatures needed for physical or chemical procedures, influence the environment and health, making them unsuccessful techniques [5, 6]. Biosynthesis techniques, on the other hand, are preferred over all methods since they frequently take only one green step and are inexpensive, secure, and clean; also, the biosynthesized nanomaterial have more precisely defined sizes and shapes [7–9].

Nanoparticles produced by microorganisms, especially fungal-based ones, are being carefully examined for novel characteristics. The most beneficial method for generating various nanomaterials has brilliantly been revealed as fungi-mediated biosynthesis [10]. Fungal species are selected broadly due to the capability of producing highly stable nanoparticles, thus preventing accumulation and increasing immortality [11]. Fungi often exhibit more creativity than bacteria in synthesizing nanoparticles as they contain a variety of bioactive metabolites and increase production [12]. Synthesis of different fungus-coated nanoparticles has been recently examined [13–15].

As a crucial metal, AgNPs have various uses, including catalysis, electronics, medical diagnosis, and antimicrobial efficiency [16, 17]. Additionally, they exhibit properties including anticoagulant properties, diabetic and thrombolytic effects, and anti-proliferative action against cancer cells [6, 18, 19]. Silver nanoparticles produced using microbial action have notable solubility, longevity, elevated productivity and antimicrobial features. The production of AgNPs is also inexpensive and harmless [15].

Nanoparticles’ antibacterial properties Compared to antibiotics have a range of mechanisms affiliated with them. Additionally, The activity of nanoparticles is significantly influenced by their surface area. However, it differs depending on the type of nanoparticles. Silver’s antibacterial activity is currently widely established [20], nevertheless, studies on silver nanoparticles (< 100 nm) have developed as a result of its multifaceted employment and the excellent defense against many bacteria, including Gram-negative and Gram-positive types [21].

Biosynthesized AgNPs have been discovered to have multiple functions with potential therapeutic effectiveness to manage harmful microorganisms [22]. Also, AgNPs’ cytotoxicity and anticancer potential were assessed in opposition to ‘HT-29’ cell lines [23], ‘MCF-7’ breast cancer cell line [24], ‘Hep2’ cell line [25] ‘MCF-7’ and ‘A549’ cancers of the breast and lungs cells, respectively [26], and Human cervical cancer cell [27].

In recent studies, metallic nanoparticles have been produced using extracts from various fungal species [9, 15, 28–33], *Aspergillus templicola* has never been employed as a reducer to mycosynthesize AgNPs. Unlike prior studies using common strains (Table 1), our work introduces *A*. *templicola* OR480102 as a high-efficiency biocatalyst, yielding stable, therapeutic-grade AgNPs under eco-friendly conditions. This system uniquely combines rapid synthesis (< 1 h), dual bioactivity, and scalability absent in fungal-AgNP literature [6, 15, 34]. Here, an aqueous extract of *A. templicola* MAK 223 was used for the first time to fabricate AgNPs. Some analytical approaches have characterized the produced AgNPs, describing their morphology, structure, and chemical properties. Additionally, this work’s objective was to assess mycosynthesized AgNPs’ effectiveness in various medical applications, such as antibacterial, cytotoxicity, anticancer, and scratch assay.

**Table 1:** AgNPs producing *Aspergillus* spp and their characters, applications

Aspergillus spp	Characters	Applications	References
*A. templicola* MAK 223	Spherical shape with size 17.78 nm	Antibacterial agent, Anticancer agent	Current study
*A. fumigatus* BTCB10 (KY486782)	Cube-shape with size 0.681 nm	Antibacterial agent, Antiproliferative agents	[35]
*A. fumigatus* DSM819	spherical shape with size less than 84.4 nm	Antimicrobial agent, Antitumor agent	[36]
*A. niger*	Spherical shape with an average size of 10–100 nm	Antifungal agent	[37]
*Aspergillus sydowii*	spherical shape with an average size of 1–24 nm	Antifungal agent, Antiproliferative agent	[6]
*A. aureoles*	spherical shape with an average size of 12–15 nm	Antimicrobial agent	[38]
*A. terreus*	Spherical shape with size 14 nm	Antimicrobial agent, Antitumor agent	[34]
*A. caespitosus*	spherical, hexagonal, and anisotropic crystalline nanoparticles with an average size of 22–57 nm	Antimicrobial agent, Anticancer agent	[39]
*A. flavus* F5	Spherical shape with size 12.5 nm	Antibacterial agent, Anti-Candida agent, Acaricide, Photocatalytic agent	[40]
*A. flavus*	regular and spherical in shape with size 474.2 nm	Antibacterial agent	[41]
*A. flavus*	Spherical shape with size 41.9 nm	Antibacterial agent	[42]
*A. hortai*	Spherical shape with size 57 nm	Antibacterial agent	[43]

## Materials and methods

### Source of tested microorganisms and used chemicals

A collected soil sample from the desert (Sandy soil from Sohag Desert, Egypt) was diluted for fungal isolation (source soil pH = 9.2). Out of them, isolate MAK223 was utilized for AgNPs production which was morphologicaly identified as *Aspergillus templicola.* Fresh cultures were periodically re-cultured on potato dextrose agar (PDA) slants and stored at 4 °C. The used human pathogens: *Escherichia coli* (*E*. *coli* ‘ATCC 25922’), *Salmonella typhimurium* (*S*. *typhimurium* ‘ATCC 14028’), *Bacillus subtilis* (*B*. *subtilis* ‘ATCC 6633’), and *Staphylococcus aureus* (*S*. *aureus* ‘ATCC 25923’) were purchased from American Type Culture Collection ‘ATTC’. Silver nitrate ‘AgNO_3_’ was purchased from Sigma-Aldrich.

### Identification of tested fungal isolate: as shown in Fig. (1a)

#### Chromosomal DNA extraction

Isolated fungus (MAK223) was cultured (used triplicate cultures) in 20 mL PD broth medium for 6 days on an incubating rotary shaker (28 °C) rotating at 250 rpm. The formed pellets were separated by centrifugation (4 °C) at 5,000 rpm, for 20 min, twice. A wight of 700 mg of the formed pellets was transferred into clean microfuge tubes and washed twice with 500 µL sterile purified H_2_O to remove any cell-attached constituents of the medium, finally, the pellet was stored at − 80 °C for one day. After exposing the sample to liquid nitrogen for five seconds, the frozen mycelium was pulverized with a sterile pestle to aid in the lysis of the fungal cell wall. Deoxyribonucleic acid (Genomic DNA) was extracted using an UltraClean^®^ Microbial DNA Isolation Kit from Mo Bio Laboratories, Inc., USA, following the guidelines provided by the manufacturer.

#### PCR amplification of the small subunit (SSU)

Using the forward primer 18SF (5^/^-GACTACGACGGTATCTAATC-3^/^) and the reverse primer 18SR (5^/^-TTAAGCCATGCATGTCTAAG-3^/^), the PCR was used to obtain the 18 S rRNA gene from pure genomic DNA. The PCR was conducted using the Qiagen Proof-Start Tag Polymerase Kit (Qiagen, Hilden, Germany). In a total volume of 25 µL, the following materials were combined: 0.5 µL genomic DNA (100 ng), PCR Master Mix (12.5 µL), each primer (0.5 µL; 5 pM), and the final volume of the reaction was reached by adding H_2_O free DNAase (11.5 µL). The previous mixture was incubated in an automated heat cycler (Master cycler, Eppindorff, Germany). Thus, to complete the assembly of all strands, there is a preliminary denaturation stage at 94 °C for 30 s, an annealing step at 52 °C for 30 s, and a final prolongation step at 72 °C for 3 min. Cold treatment at 4 °C halted the reactions. Products of PCR were separated by electrophoresis on an agarose gel (1%; (v/v) in a running buffer of Boric acid EDTA tris-base (TBE). The gels were then examined under ultraviolet (UV) light. The QIA rapid gel separation kit (Qiagen Inc, Hilden in Germany) separated the PCR materials, which had a bp value of approximately 1100, from the gel.

#### Sequencing of DNA

The PCR products, after amplification, were shipped to Macrogen in Seoul, South Korea, for bidirectional sequencing via an automatic Genome sequencer (Applied Biosystems^®^, Thermo Scientific Fisher, Foster City, Calif.; 3500 series Genetic Analyzer). The resulting sequences were compared with deposited sequences accessible through http://www.ncbi.nlm.nih.gov/BLAST/ (BLAST search on the National Centre for Biotechnology Information) website to evaluate DNA similarity and verify identification. Several sequence analyses were conducted, and the phylogenetic tree of MAK 223 was constructed [44].

### Mycogenesis of AgNPs by *A. templicola* filtrate: as shown in Fig. (1b)

*Aspergillus templicola* MAK223 (3 independent batches) was incubated into PDB over 10 days at 28 ± 2 °C, with regular observation of fungal growth. Next, the mycelium was filtered over Whatman paper filters (Grade 1) to eliminate the residual media, the filtered mass underwent three washes with distilled water [15]. Subsequently, ten grams of fungal mycelia (fresh weight) were added into 100 mL sterile purified H_2_O and were incubated on a rotary shaker (120 rpm) at room temperature for 72 h. The resulting mixture was then filtered using the same type of filter paper. Finally, filtrate (9 mL) was mixed with AgNO_3_ (1 mL, 1 mM), and this mixture was incubated at room temperature for one day in dark. Control samples (without adding AgNO_3_) were included for comparison.

### Characterization of the mycosynthesized agnps: as shown in Fig. (1c)

#### UV-Vis spectroscopy measurements

The first indication of AgNPs production was detected visually by noticing the change in color shifting into brown and confirmed using a JENWAY 7315 spectrophotometer (UK) for UV-vis measurements. The instrument is adjusted to scan the absorption from 300 to 700 nm.

#### Transmission electron microscopy analysis

Transmission electronic microscopy (TEM) (JEOL-JEM-100CX II) was used to visualize the biosynthesized AgNPs’ initial size and shape using micro-drop coating method, where a drop (5 µL) of AgNPs’ solution was placed on the copper grids (carbon coated) and dried overnight at room temperature before being loaded into the sample holder [45]. Using micrographs, the AgNPs’ size distribution was assessed. A size distribution histogram was then utilized to determine the average size of the generated AgNPs. Ninety-six nanoparticles were measured and statistically analyzed using the Gaussian fit confirmed with R²=0.98 (OriginLab; version 2019b).

#### Fourier-transform infrared (FT-IR) spectroscopy

Various kinds of functional groups (-NH, C = O) and polysaccharides (-OH) of both fungal filtrate and AgNPs biosynthesized using *Aspergillus templicola* MAK223 filtrate are utilized to stabilize and cap mycosynthesized AgNPs using FT-IR spectroscopy (Germany, ALPHA II, platinum ATR). Characteristic peaks were recorded in 400–4000/cm at a resolution of 4/cm [46].

#### Dynamic light scattering

Before measurement, the sample was sonicated for three minutes. Then, the analyzed AgNPs solution was assessed for size and distribution, represented by average volume diameters and polydispersity index. This analysis was performed using Dynamic light scattering (DLS) with an analyzer of particles’ size (Zetasizer Nano ZN, Malvern Panalytical Ltd, UK) at a fixed angle of 173, and the measurements were conducted at 25 °C, via identifying dynamic shifts in scattered light intensity brought on by the particles’ Brownian motion, the results showed the particles’ standard hydrodynamic diameter, the hydrodynamic dimension dispersion peak values and polydispersity index (PdI) that indicated the particle size distribution’s width. The samples were examined in triplicate, employing the same equipment to assess zeta potential. PdI values range from 0 to 1 (monodisperse at 0 and polydisperse at 1).

### Optimum mycosynthesis conditions of AgNPs

Fungal- mediated silver nanoparticles (AgNPs) were optimized by studying four main factors: the incubation periods, the concentration of the silver nitrate (AgNO3) substrate, pH different values, and different temperature degrees. Each factor was changed individually, while the others remained the same. The incubation periods were 3, 5, 7, 10 and 13 days. The AgNO_3_ concentrations were tested at 0.25, 0.5, 1, 2, and 3 mM. The pH was adjusted to 3, 5, 7, 9, and 11 using 0.1 N HCl or 0.1 N NaOH solutions. The temperatures were tested at 30, 40, 60, 80, and 100 °C. Each factor was studied by following the highest absorption peak in UV-visible spectroscopy.

For increasing concentration of collected AgNPs for biological application, the fungal filtrate was prepared by suspending 50 g of fungal biomass in 500 mL of sterile distilled water. After filtration and centrifugation, the supernatant was reacted with 1 mM AgNO₃ solution at pH 11 for 10 days. The resulting AgNP suspension was centrifuged (15,000 rpm, 20 min) and washed sequentially with ethanol (3×) and distilled water (3×). The purified AgNPs were dried at 60 °C for 1 h and resuspended in sterile water to prepare stock solution. So all required concentrations can be prepared through serial dilution of the stock one for biological testing.

### Mycosynthesized AgNPs activities: as shown in Fig. (1d)

#### Sterility test

Beforehand, a sterility test of 20 mL (80 µg/mL) of formed AgNPs in cell free filtrate of *A. templicola* MAK223 was performed. The solution passed through a sterile syringe filter (0.22 μm). Two types of media were used: fluid thioglycolate medium (FTM) for anaerobic and aerobic bacteria and tryptic soy broth (TSB) for fungi and aerobic bacteria. Both culture media were prepared according to manufacturer instructions and then autoclaved. A 1.5 mL of AgNPs solution was used to inoculate each medium (100 mL) and incubated at 32.5 ± 2.5℃ and 22.5 ± 2.5℃ for FTM and TSB, respectively for 7 days. Uninoculated media (100 mL) were served as negative control and subjected to identical incubation circumstances.

#### Antibacterial activity of AgNPs

The antibacterial effectiveness was performed in compliance with the Laboratory Standard Institute of Clinical and Health instructions [47] as follows:

##### Preparing inoculum (colony suspension Method)

To achieve overnight (16–18 h) cultures at 37 °C ± 1.0, fresh colonies of pathogenic Gram-negative and Gram-positive bacteria (*E*. *Coli* ‘ATCC 25922’, *S*. *typhimurium* ‘ATCC 14028’, *B*. *subtilis* ‘ATCC 6633’, and *S*. *aureus* ‘ATCC 25923’) were cultured in 10 mL Luria Broth (LB), except for *B*. *subtilis* which was incubated at 30 ℃ ± 1.0. Suspensions were calibrated using the DensiCHEK© optical instrument to turbidity that matches each strain’s 0.5 McFarland standard at a wavelength of 600 nm. By introducing 1 mL of inoculum into 150 mL of Muller Hinton broth (MHB), suspensions were diluted (~ 1.0 × 10^6^ colony forming unit per milliliter. Hence, any subsequent 1:2 dilution will result a 5.0 × 10^5^ CFU/well.

##### Broth macro Dilution method

In a Corning™ Costar™ Flat Bottom Cell Culture Microplates (24 wells) 1.0 mL of Muller Hinton broth (MHB) was applied to all testing wells except the first one. Where, the first well is loaded by undiluted 2 mL AgNPs (80 µg/mL). Then a subsequent dilution of AgNPs was made by transferring 1 mL from the first well to the next one (1.0 mL MHB + 1.0 mL AgNPs) and mixing well by pipetting. This step is repeated in the following wells to get nine AgNPs concentrations (40, 20, 10, 5, 2.5, 1.25, 0.625, 0.3125, and 0.156 µg/mL).

After that, an inoculum of 1 mL of each bacterial broth was used for inoculating the tested wells (final concentration 5.0 × 10^5^ CFU/well). To verify inoculum density, 1.0 mL of each bacterial suspension was simultaneously diluted and cultivated (externally). Controls (Positive and negative) were prepared in each sample per plate. Broth was the only negative control, and gentamicin was tested against all bacterial strains, which was the positive control. Also, a growth control was performed in a well containing the broth and the bacteria without AgNPs. For twenty-four hours, each microplate was incubated at 36 °C ± 2, except for *B*. *subtilis*, which was incubated at 30 ℃± 1. Determining the minimal inhibitory concentration (MIC) was achieved by finding a concentration level that, in comparison to the growth control, suppresses ≥ 80% of growth.

A cork borer was used to create 4 wells (6 mm) to detect clear zone formation. For comparison, fungal filtrate, AgNO_3_ (0.5 mM), AgNPs (80 µg/100 µL), and Gentamicin (80 µg/100 µL) were added individually to each well of the Petri plates. Ultimately, all plates were stored for a full day at 37 ± 2 ℃ before being investigated. For every bacterial strain, this experiment was conducted in triplicate (each strain was tested in three independent experiments with triplicate wells per concentration).

#### Cell cycle test of ‘MCF-7’ using AgNPs

##### Cell culture Preparation

The ‘MCF-7’ cell cycle distribution was evaluated by measuring DNA contents according to the fluorescence intensity using 3 biological replicates. Modified Eagle Medium (DMEM) from Dulbecco is utilized to encourage the development of ‘MCF-7’ breast cancer cell lines [48]. After heat-inactivation, 10% of fetal bovine serum (FBS) is added to DMEM. Furthermore, a concentration of 80 mg/mL of streptomycin and 80 units/mL of penicillin were provided. Cultures kept at 37 °C in a carbon dioxide incubator (5% CO_2_) (Heracell^®^ 150i / 240i GP, Thermo Fisher Scientific Inc., USA).

##### Assay for flow cytometry

Briefly, at 48 h of treating ‘MCF-7’ cells with AgNPs (50 µg/mL), trypsinization was used to pellet cells (1 × 10^5^ cell/mL), which were then twice-washed with ice-cold phosphate buffer saline (PBS; pH 7.4) and fixed for 1 h at 4 °C in 2 mL of ice-cold ethanol (60%). The result cells were washed with neutral PBS (pH ~ 7.4), centrifuged at 3,000 rpm, then re-dissolved in 1 mL PBS supplemented with 50 µg/mL RNase and 10 µg/mL phycoerythrin (PE)-Texas Red-A propidium iodide (PI) for DNA staining for 1 h in dark conditions at 37 ℃. Next, cells are subjected to flow cytometry examination with a FL2 (λex/em 535/617 nm) signal detector (ACEA NovocyteTM flowcytometer, ACEA Biosciences Inc., the California city of San Diego, USA) to determine the content of genetic material in the cells. Twelve thousand events for every sample were acquired. The ACEA NovoExpressTM programme (ACEA Biosciences Inc., the California city of San Diego, USA) is used to get cell phase distribution.

#### Wound healing assay

The wound healing test, commonly known as the scratch assay, is a method that is frequently employed to assess cell migration and wound closure kinetics. In this assay, a scratch is created on a cell monolayer, simulating a wound, and over time, the passage of cells to close the gap is monitored (3 independent experiments). By treating the cells with AgNPs, the impact on the wound closure rate can be evaluated. For cell migration monitoring, ‘MCF-7’ breast cancer cells were seeded (2 × 10^5^ cells/well) in a 12-well Collagen I coated microplate. Stored for a night at 37 °C (5% CO_2_) in 5% FBS-DMEM. At 24 h of incubation, the plate is thoroughly washed with phosphate-buffered saline (PBS); while treatment wells were filled with new media, control wells were refilled with it and fortified with 30.67 µg/mL drug (cisplatin, positive control and AgNPs, separately). Images were captured using a LABOMED inverted microscope (TCM 400 Labo America, Inc, USA) every 4 h (0, 24, 48, and 72 h) (0, 24, 48, and 72 h). The plate was incubated at the same conditions mentioned earlier in-between each time interval. The MII ImageView programme (version 3.7) was used to analyze the pictures.

### Analytical statistics

The data demonstrates the average, standard deviation (± SD), and three replicates (*n* = 3). Data analysis was done with IBM SPSS (ver. 21), Microsoft Excel 2013, and GraphPad Prism version 9.5.1. The two-tailed t-test was used to generate P*-*values, and a significance level of *P* < 0.05 was applied.

## Results

In the present study, the *Aspergillus templicola* strain MAK223 was tested for AgNPs production and the optimum conditions for their production were studied. General characters of produced AgNPs were detected in addition to their bioactivity.

The *Aspergillus templicola* strain MAK223 was molecularly identified by determining the amplified 18 S rRNA gene’s nucleotide sequence, which demonstrated that this isolate is identical to *A. templicola* strain CBS 138,181(OL 711823.1) and *A*. *templicola* strain DTO 267-H4 (KP987081.1) with 100.00% similarity. A phylogenetic tree is constructed as shown in with a branch length equals 0.200 (Fig. 2). Thirteen nucleotide sequences were examined. Sequences’ terminals of missing data were eliminated. The 18 S rRNA nucleotide sequence of *A*. *templicola* MAK 223 was registered into the GenBank database with ‘accession number’ OR480102.

**Fig. 1:**
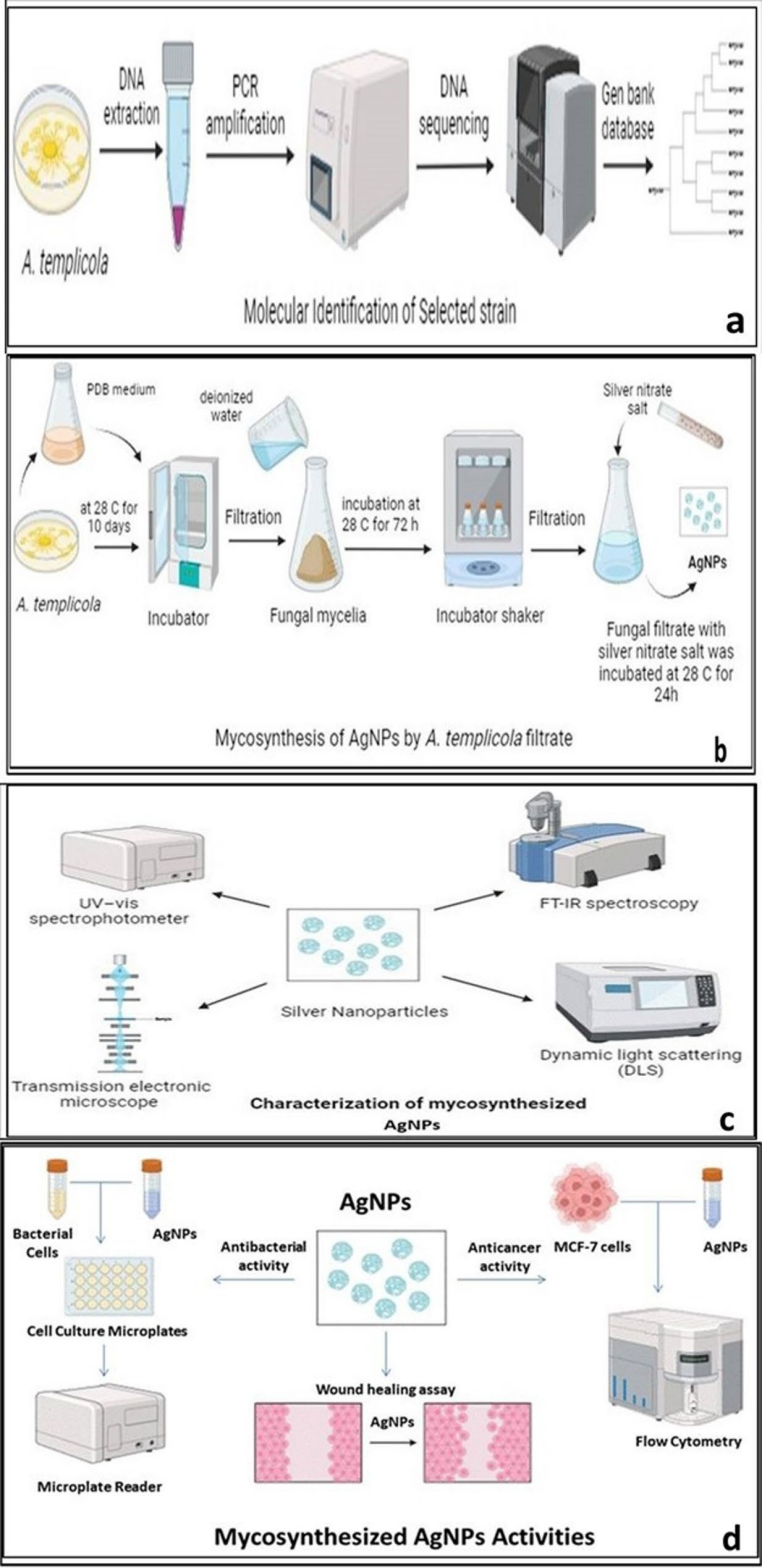
Experimental design presented in this study to demonstrate molecular identification of selected strain, mycothensysis of AgNPs by *Aspergillus templicola* OR480102, their characters and activities

**Fig. 2:**
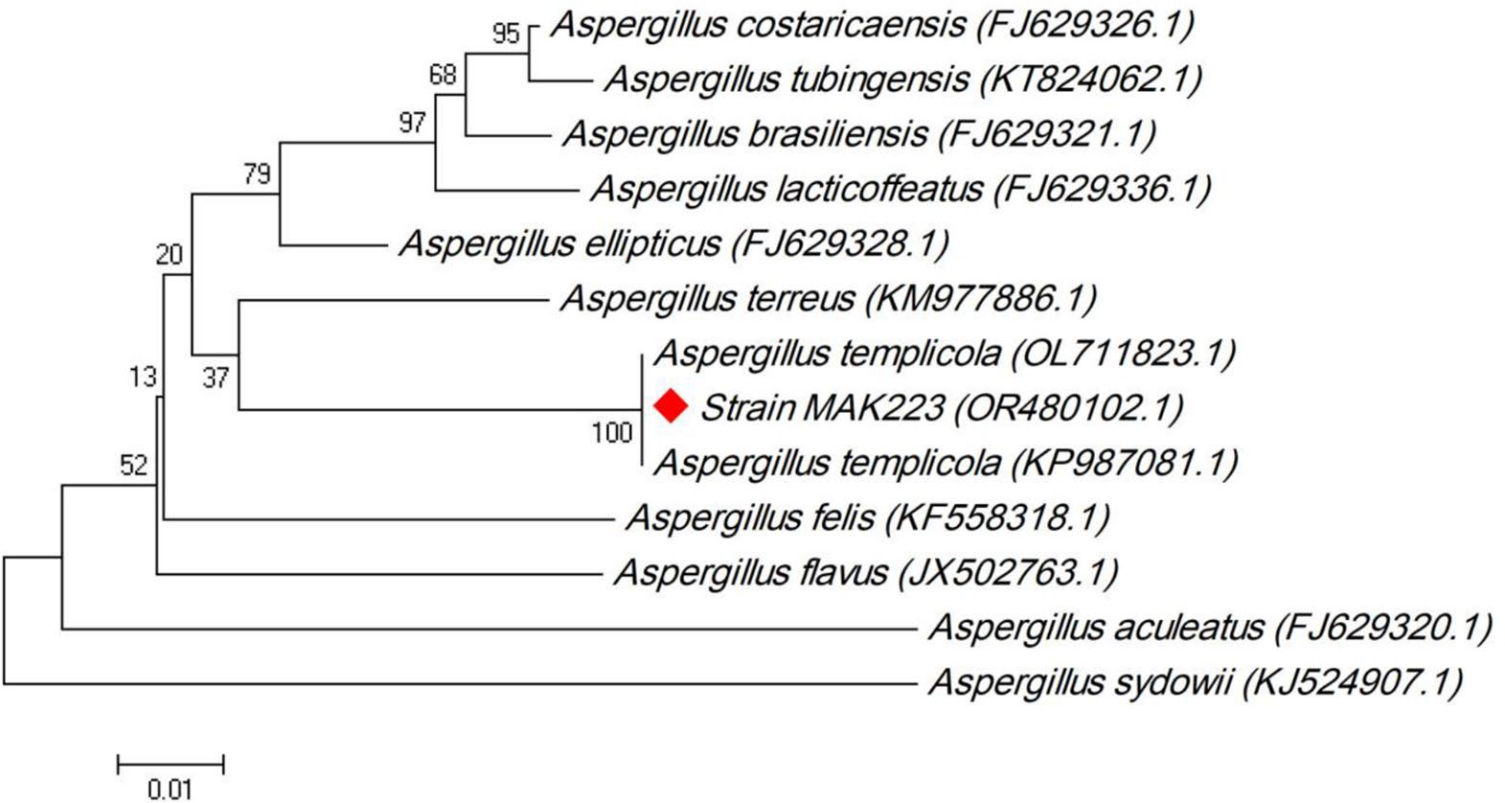
Evolutionary relationships of *Aspergillus templicola* (OR480102) with the other related *Aspergillus* strains in GenBank database

### Physical characterization of the biogenic AgNPs

**Fig. 3:**
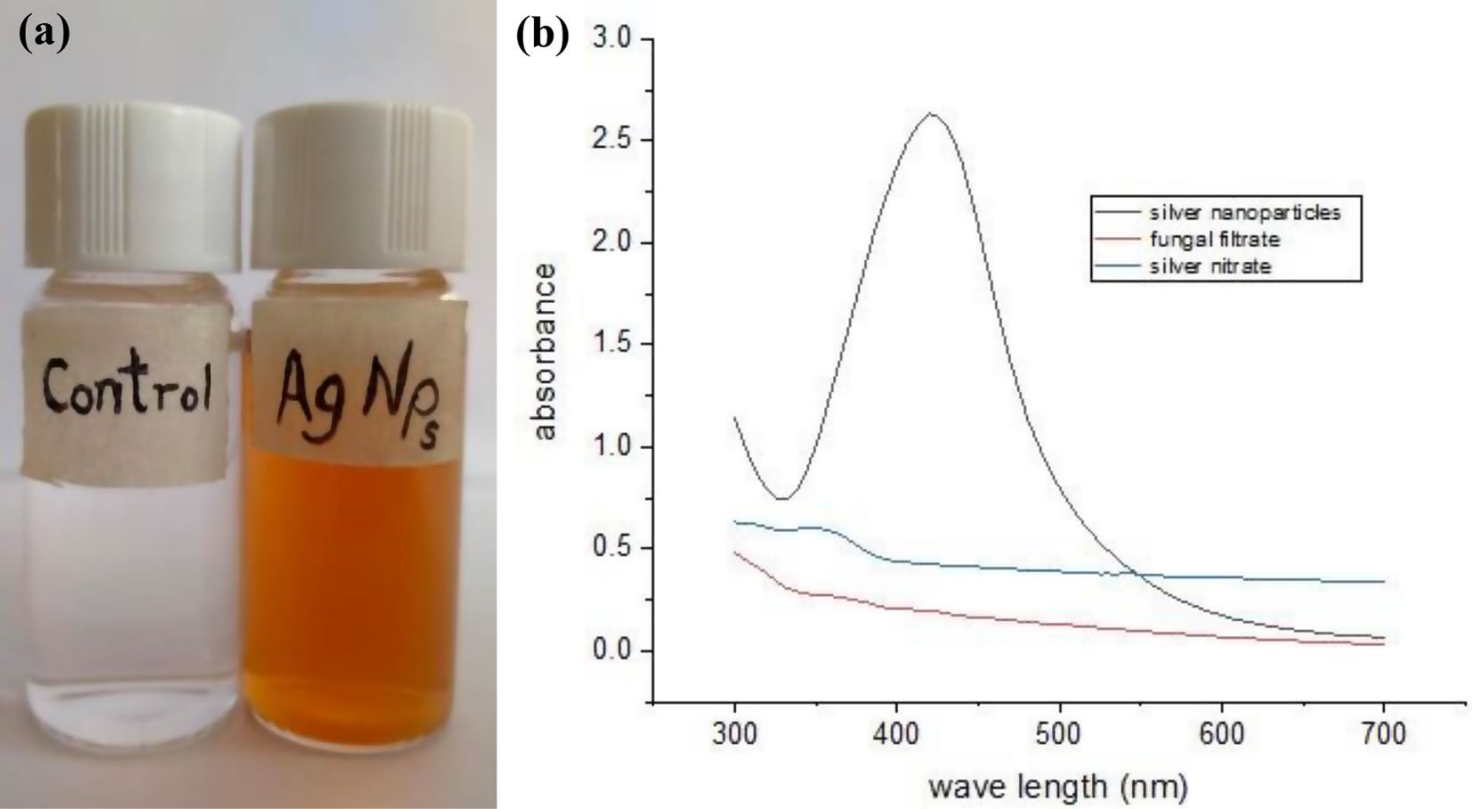
UV–visible absorption spectrum of AgNPs biosynthesized by *Aspergillus templicola* MAK 223

AgNPs were biosynthesized by reducing silver nitrate with *A*. *templicola* OR480102 filtrate, confirmed by a visible color change to brown (Fig. 3a). After incubation with silver nitrate, the silver ions (Ag^+^) were reduced biologically and a noticeable brown color demonstrating surface-plasmon resonance of metallic AgNPs. When the produced AgNPs were analyzed using UV-vis confirmed AgNP synthesis (λmax = 420 nm, Fig. 3b).

**Fig. 4:**
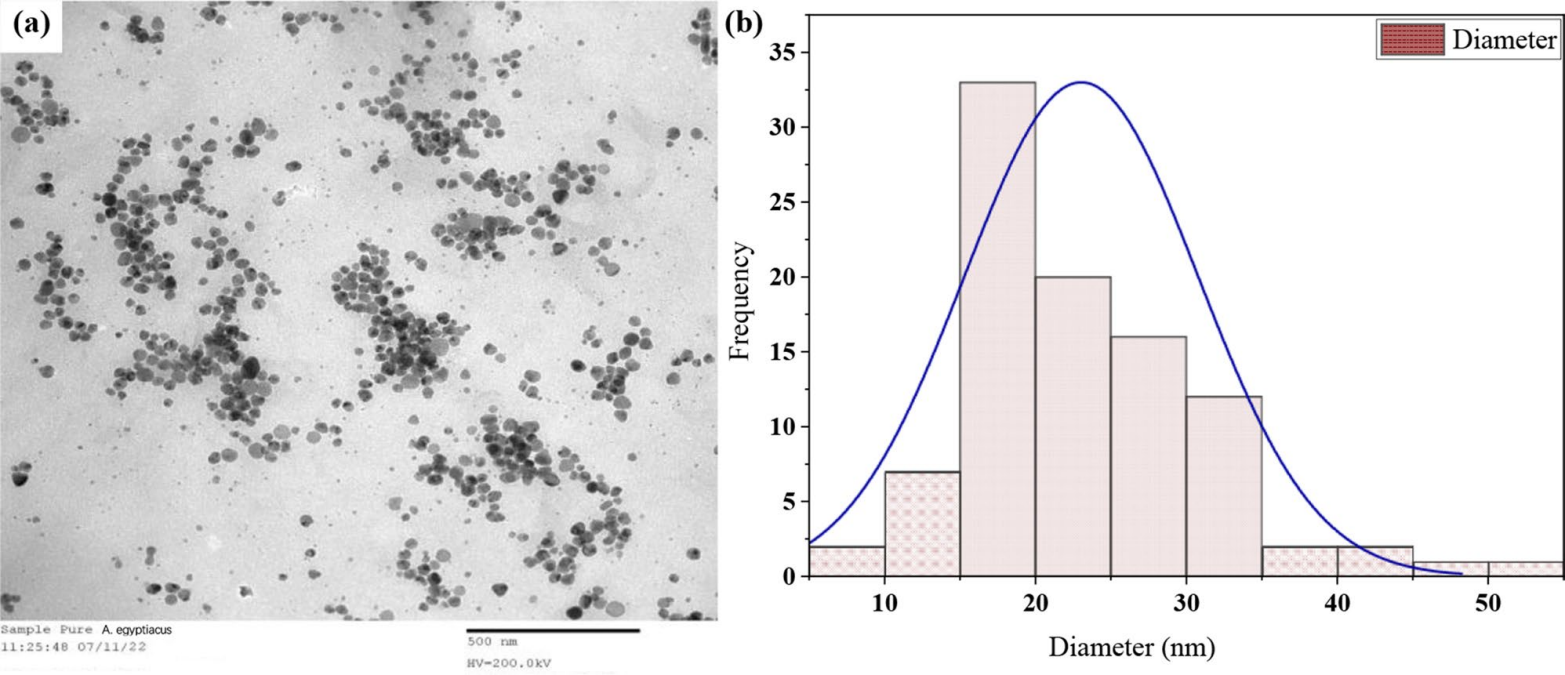
TEM image of spherical AgNPs between 5–25 nm size with average size of 17.79 ± 1.36 nm (scale bar = 500 nm); Gaussian fit confirmed with R²=0.98 (OriginLab)

The resulting nanoparticles’ size and shape are depicted in the representative TEM image (Fig. 4a), clearly demonstrating their spherical shape. Based on the size dispersion histogram, the nanoparticles had a diameter ranging from 5 to 25 nm., with mean size of 17.79 ± 1.36 nm (Fig. 4b).

**Fig. 5:**
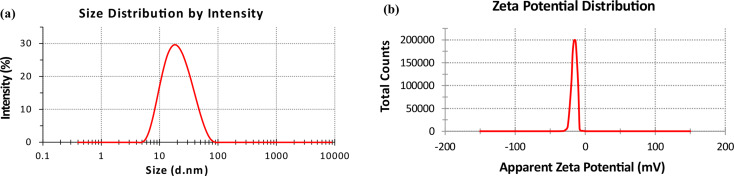
Size distribution of AgNPs solution synthesized by filtrate of *Aspergillus templicola* MAK 223 (10% gm/mL). (**a**) dynamic light scattering analysis. (**b**) zeta potential analysis

Dynamic-light-scattering (DLS) analysis is used to quantify the dynamic diameter of mycosynthesized AgNPs (Fig. 5a). The polydispersity Index equal (PdI) 0.074 indicated that AgNPs were stable. The value of PdI larger than 0.5 suggests that the NPs are less stable and have a greater chance of aggregating. Values less than 0.05 are infrequently observed in the dimensionless Polydispersity Index, in contrast to that of very monodisperse standards.

Additionally, zeta potential analysis has been used to assess the steadiness of the produced AgNPs (Fig. 5b). The surface of AgNPs showed recording charges in our examination. The − 16.5 mV zeta energy potential was an excellent negative value for AgNPs.

**Fig. 6:**
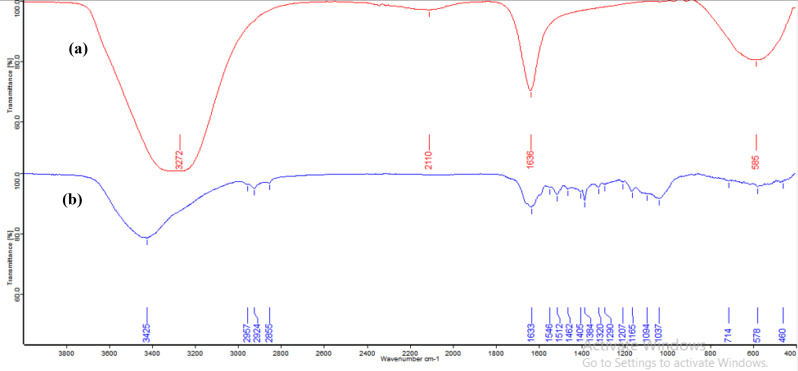
Fourier Transform Infrared (FT-IR) spectra of (**a**) *Aspergillus templicola* MAK 223 filtrate and (**b**) Mycosynthesized AgNPs

The FT-IR spectrum (Fig. 6) indicated the presence of various functional groups, including hydroxyl groups, amide I and II from proteins, NH stretching, phenolic or tertiary alcohol–OH bending, and aromatic C-H and S-O stretching from sulfonates. The fungal filtrate spectrum displayed prominent peaks at 3272 cm⁻¹, 2110 cm⁻¹, 1635 cm⁻¹, and 995 cm⁻¹ (Fig. 6a). In the spectrum of the synthesized AgNPs (Fig. 6b), the broad peak at 3272 cm⁻¹ from the fungal filtrate shifted to 3283 cm⁻¹. The peak at 2110 cm⁻¹ showed a decrease in intensity, while the C = O stretching peak at 1635 cm⁻¹ also experienced a slight shift and a reduction in intensity. The C-H bending vibration at 995 cm⁻¹ remained prominent, indicating that certain features were retained after nanoparticle synthesis.

### Optimizing fungal-mediated synthesis of silver nanoparticles

This study investigated the factors influencing the production of silver nanoparticles (AgNPs) using fungal-mediated methods. By systematically altering incubation time, silver nitrate concentration, pH, and temperature, we determined the optimal conditions for AgNP synthesis.

**Fig. 7:**
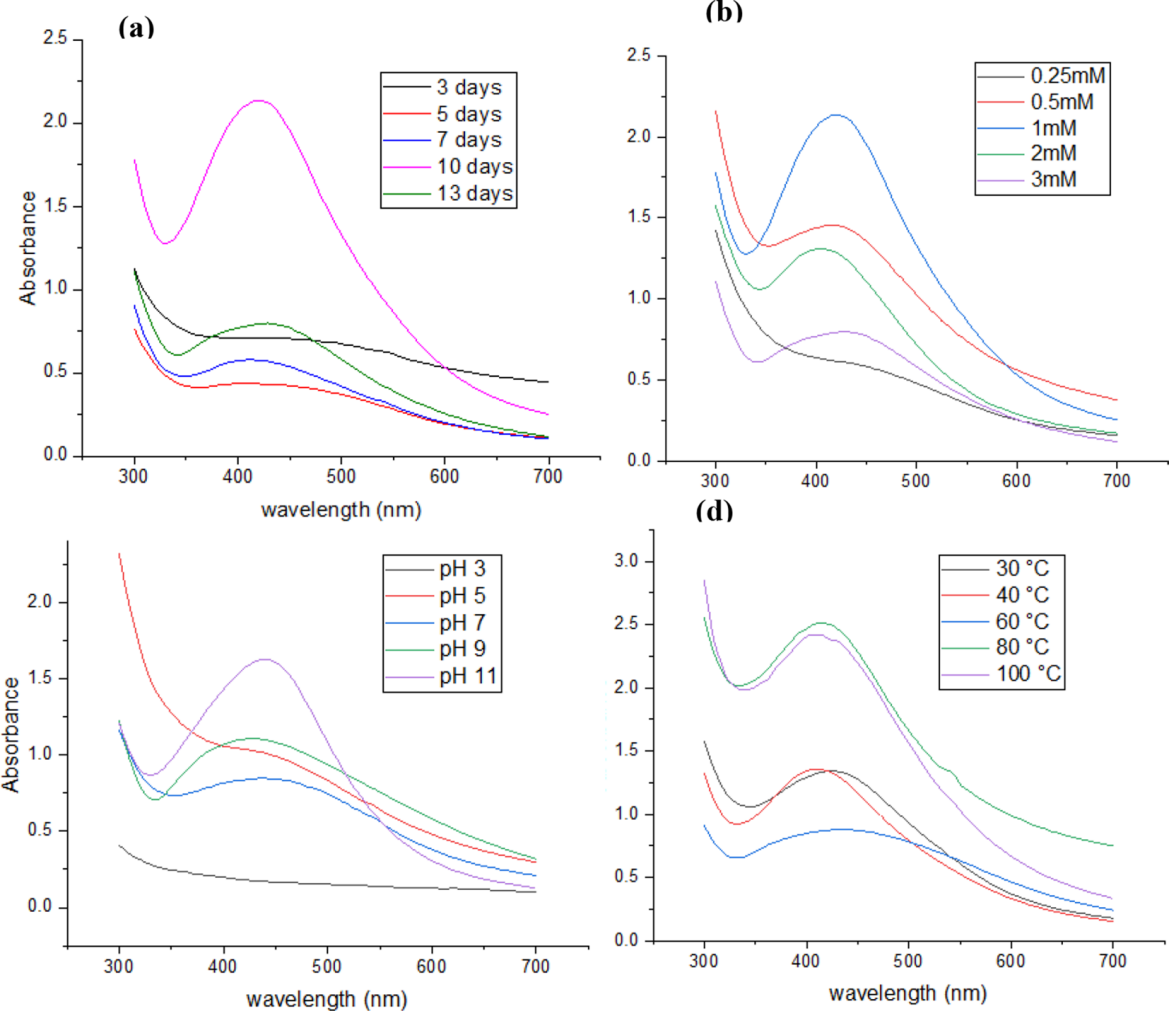
Optimum reaction conditions of AgNPs biosynthesized using *Aspergillus templicola* MAK 223 filtrate using (**a**) Different reaction periods, (**b**) Different AgNO_3_ concentrations, (**c**) Different pH values, and (**d**) Various temperature degrees

Prolonged incubation periods have resulted in the highest yield and stability of AgNPs. Ten days of incubation proved the ideal duration (Fig. 7a). A concentration of 1 mM silver nitrate was the most effective for AgNP formation. Lower concentrations led to reduced nanoparticle production, while higher concentrations resulted in aggregation and inconsistent particle size (Fig. 7b). A highly alkaline environment (pH 11) was optimal for AgNP synthesis. Lower pH values hindered the reduction of silver ions, while higher pH levels promoted nanoparticle formation and stability (Fig. 7c). Elevated temperatures significantly accelerated AgNPs synthesis. This is attributed to the increased kinetic energy at higher temperatures, which facilitates the reduction of silver ions (Fig. 7d). The 500 mL fungal filtrate yielded 40 mg of AgNPs from 500 mL fungal filtrate (80 µg/mL stock concentration). This stock solution was diluted to generate all test concentrations.

### Antibacterial activity of AgNPs

**Fig. 8:**
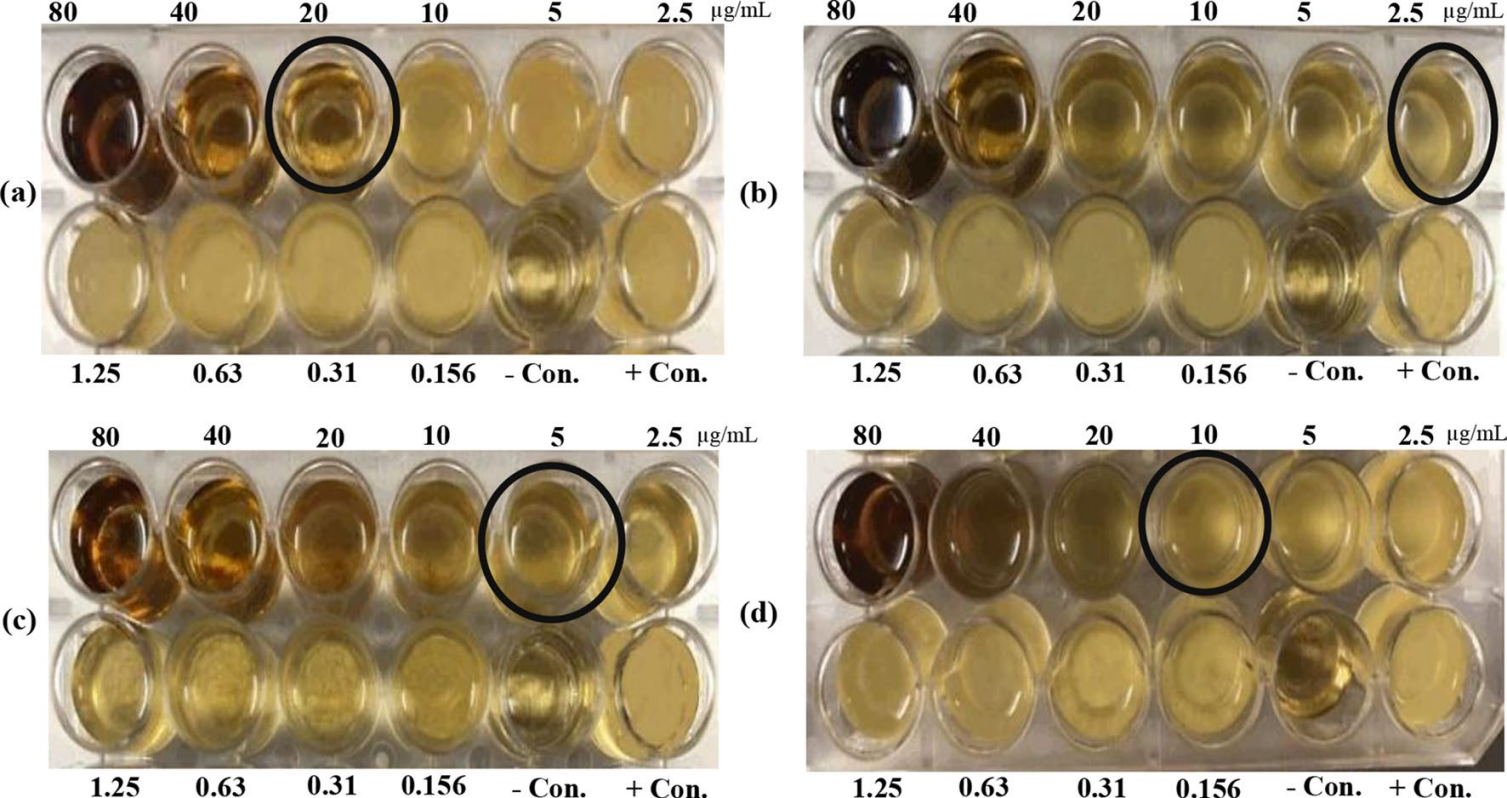
Minimal inhibitory concentration (MIC) of AgNPs against four bacterial strains. Gram-negative bacteria: (**a**) *Escherichia coli* ATCC 25,922 and (**b**) *Salmonella typhimurium* ATCC 14,028. Gram-positive bacteria: (**c**) *Bacillus subtilis* ATCC 6633 and (**d**) *Staphylococcus aureus* ATCC 25,923

**Fig. 9:**
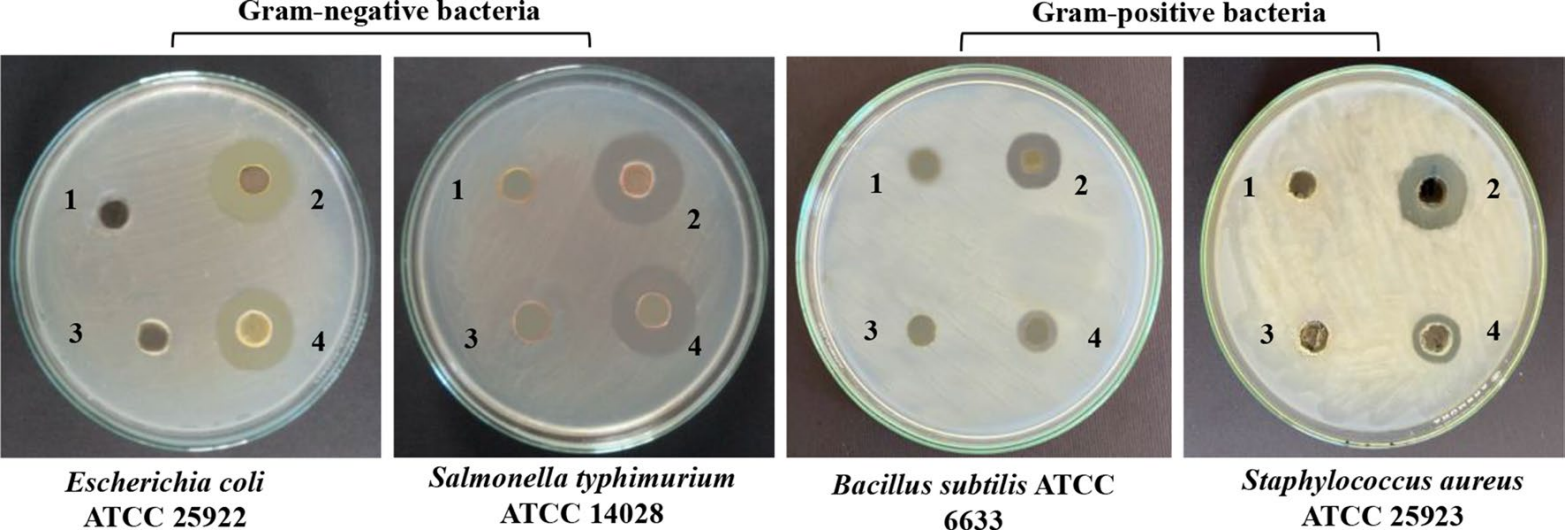
Antibacterial test of AgNPs produced by *A*. *templicola* filtrate. 1 = cell-free fungal filtrate, 2 = 80 µg AgNPs, 3 = 0.25 mM AgNO_3_, and 4 = 80 µg gentamicin)

The recorded MIC for *E*. *coli* ‘ATCC 29213’, *S*. *typhimurium* ‘ATCC 14028’, *B*. *subtilis* ‘ATCC 6633’, and *S*. *aureus* ‘ATCC 25923’ were, 20, 2.5, 5, and 10 µg/mL, respectively (Fig. 8; Table 2). Measured clear zones (mm) of synthesized AgNPs were equal to the size of positive control or higher against Gram-negative bacteria (Fig. 9; Table 2). Also, AgNPs exhibited more inhibition for Gram-positive bacteria than the positive control. Neither the cell-free fungal extract nor the AgNO_3_ control showed any inhibition for the growth of all tested bacteria.

**Table 2:** Clear zone (mm) and MIC values (µg/mL) of AgNPs against four pathogenic bacteria

Bacteria		MIC (µg/mL)		Inhibition zone diameter (mm)	
		AgNPs	Gentamicin	AgNPs	Gentamicin
Gram-negative	*Escherichia coli* ‘ATCC 29,213’	20	10	11 ± 0.3	9 ± 0.4
	*Salmonella typhimurium* ‘ATCC 14,028’	2.5	20	13 ± 0.5	13 ± 0.3
Gram-positive	*Bacillus subtilis* ‘ATCC 6633’	5	20	5 ± 0.2	3 ± 0.6
	*Staphylococcus aureus* ‘ATCC 25923’	10	10	8 ± 0.3	3 ± 0.5

### Evaluation of AgNPs effect on ‘MCF-7’ cell apoptosis using flow cytometry

**Fig. 10:**
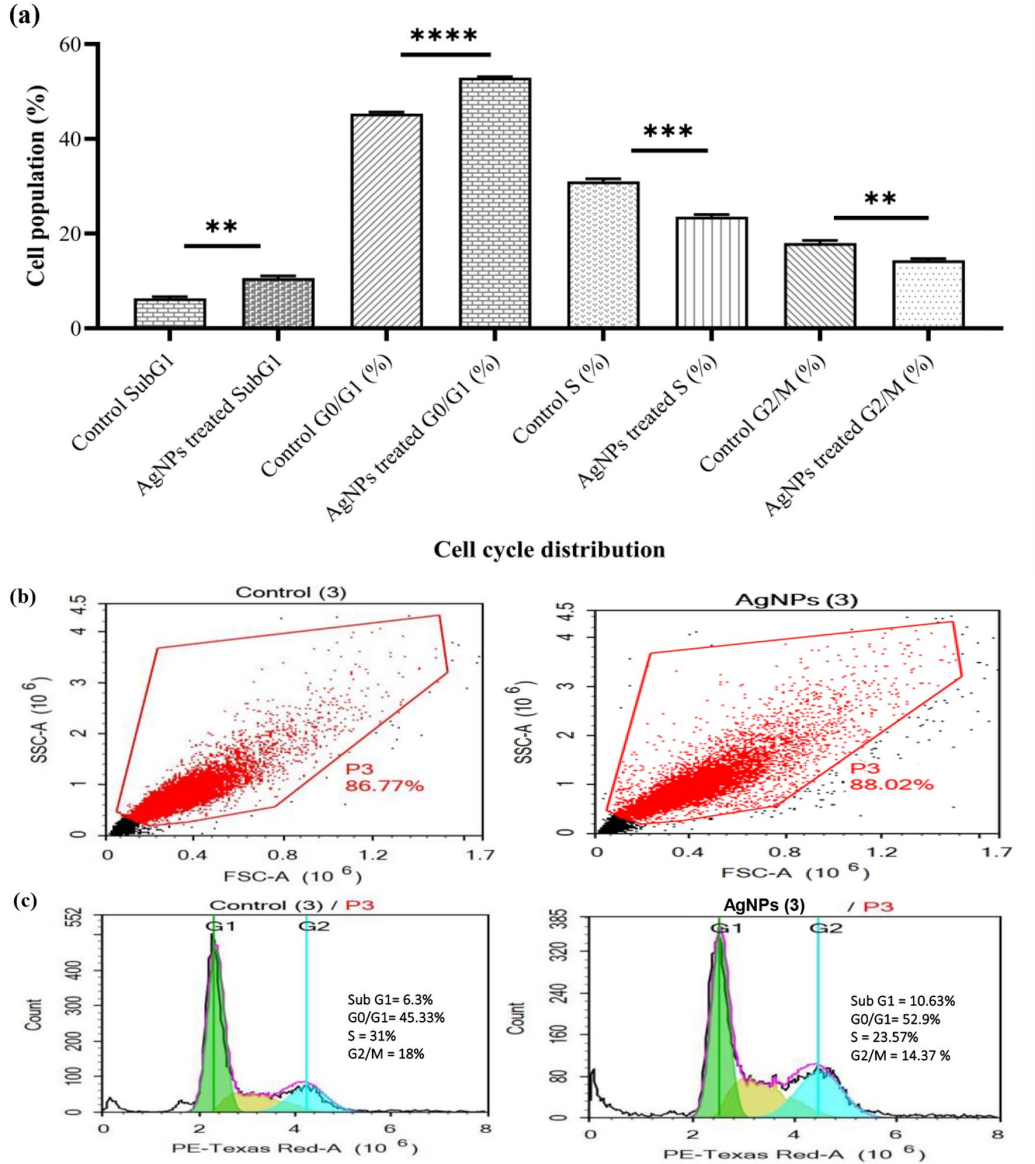
Flow cytometry analysis of control ‘MCF-7’ cell line and AgNPs-treated cells at 48 h. (**a**) Bar chart of cell cycle distribution and percent of DNA content in each phase, the results are presented as mean ± standard deviation of three replicates in three independent tests; an asterisk * indicates statistically significant difference from untreated control (*P* < 0.05; where, ** = P value 0.0017, *** = P value 0.0006, and **** = P value < 0.0001); (**b**) 2D scatter plots (FSC vs. SSC); and (**c**) DNA histogram displays four phases of cell cycle distribution

MCF-7 cells’ viability showed inhibition when handled with prepared AgNPs at IC_50_ (50 µg/mL) after 48 h (Fig. 10a, b, and c). Control ‘MCF-7’ cells typically exhibited a more significant percentage of cells in the S (cells that are undergoing DNA synthesis during replication) and G2/M (mitosis) phases.

### Wound healing assay

**Fig. 11:**
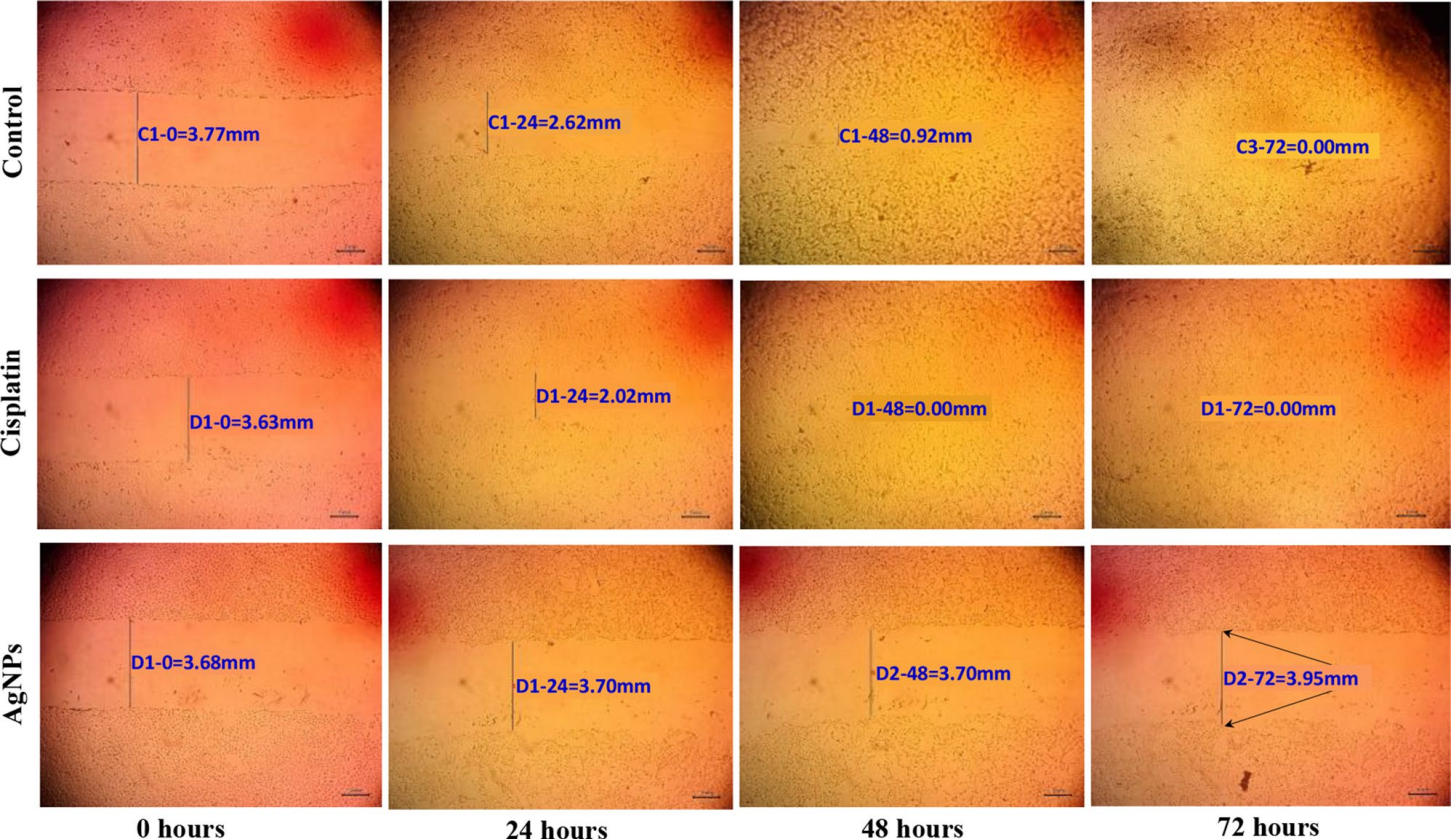
Images of wound width (mm) at four-time intervals for control, cisplatin, and AgNPs. Black arrows show unhealing of ‘MCF-7’ cells after 72 h using AgNPs

**Fig. 12:**
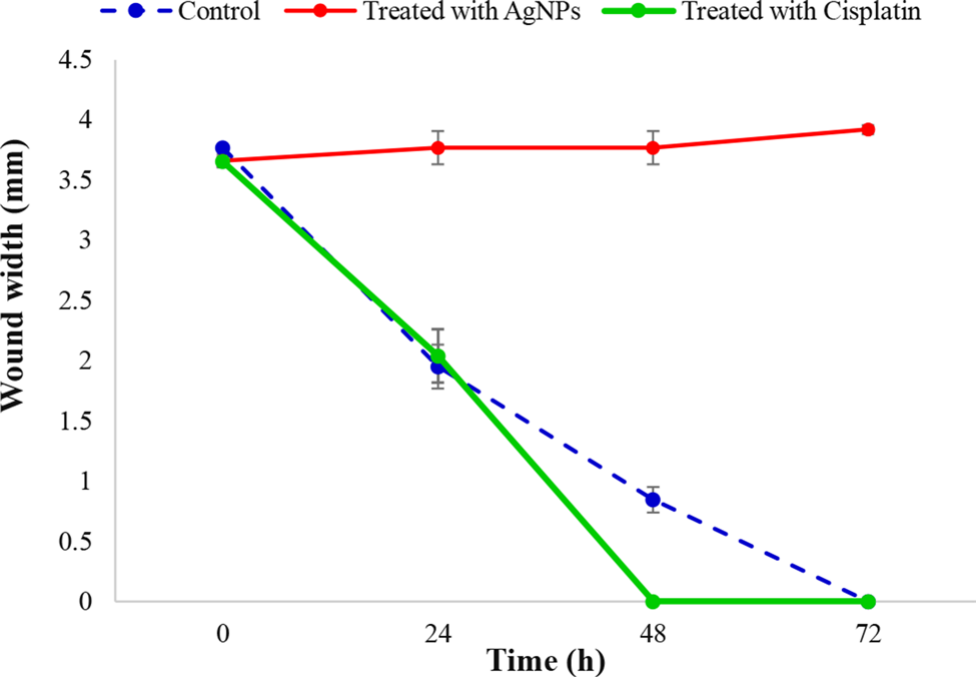
Wound healing rate. The dashed line represents wound healing rate in control cells. The red and green lines represent the wound healing rate in cells treated with AgNPs and cisplatin, respectively

Examined wound width (mm) images showed that cisplatin helped cells to cure after 48 h while AgNPs prevented the cancer cells from healing even at 72 h of treatment (Fig. 11). Results are displayed as mean ± standard deviation (Fig. 12).

## Discussion

Microbial culture-based nanomaterial synthesis is now a potential biological fabrication technique that can contribute to the advancement of more creative and environmentally friendly processed nanomanufacturing [10]. Table 1 showed different *Aspergillus* spp which used for biosynthesis of AgNPs, in addition to their characters and applications.

The *A. templicola* strain used in this investigation was given OR480102 as an ‘accession number’ and deposited into the GenBank database. Silver ions underwent bioreduction by fungal extract, and the mycosynthesised AgNPs were analyzed by applying UV–visible spectrophotometry, they exhibited a strong plasmonic maximum elevation at 420 nm. AgNPs made by *A. terreus* have their maximum UV absorption measured by Lotfy et al. [34] at 420 nm. Also, comparable results were recorded using *Penicillium chrysogenum* [49]. The strong SPR characteristic of the particles triggered the excitation at 420 nm [50]. During the present study, even after the reaction had occurred three months earlier, the solution had great stability and no signs of nanoparticle flocculation, this stability results from numerous proteins and other biomolecules secreted by the used fungus. This strain’s unique exoproteome (Fig. 6) enables both rapid reduction and natural stabilization of AgNPs—addressing two key limitations of fungal synthesis: slow kinetics and particle aggregation [34, 40].

In the TEM picture, the created AgNPs had a spherical form and averaged 17.78537 ± 1.36 nm in size., which agrees with Bhainsa and D’Souza [51] who reported biosynthesis of 5–25 nm AgNPs extracellularly using *Aspergillus fumigatus*. Furthermore, Xue et al. [52] and Gudikandula et al. [53] demonstrated the mycosynthesis of spherical 5–40 nm AgNPs using two white rot fungal strains. These results can recommend that, in contradiction to previously synthesised NPs, fungal species can produce smaller AgNPs [54].

The polydispersity Index (PdI) of mycosynthesized AgNPs equal 0.074. Masarudin et al. [55] discovered that the AgNPs sample is most likely unsuitable for DLS due to its extremely wide size distribution, with (PdI) values greater than 0.7. Additionally, the mycosynthesized AgNPs demonstrated a favorable negative zeta potential of -16.5 mV, as determined through zeta potential analysis. The values obtained from this analysis indicate the stability of the mycosynthesized nanoparticles [56]. This outcome suggests a high-quality nanoparticle aggregation. The presence of negative charges suggests that AgNPs are encased in negative biomolecules, effectively reducing repulsion between the nanoparticles, decreasing aggregation, and increasing their stability overall [32, 57].

FTIR analysis was used to identify the functional groups involved in synthesizing and stabilizing silver nanoparticles (AgNPs) with fungal filtrate. The fungal filtrate showed key peaks at 3272 cm⁻¹ (O-H stretching) [58], 2110 cm⁻¹ (alkyne groups), 1635 cm⁻¹ (C = O stretching in proteins) [59], and 995 cm⁻¹ (C-H bending) [60]. After AgNPs synthesis, shifts in these peaks, particularly at 3272 cm⁻¹ and 1635 cm⁻¹, suggest hydroxyl and carbonyl groups play roles in reducing Ag⁺ to Ag⁰ and stabilizing the nanoparticles. The biomolecules in the fungal filtrate act as both reducing and capping agents, preventing aggregation of AgNPs [61]. These results align with those from other investigations [9, 15, 62, 63].

The optimization of AgNPs synthesis through fungal-mediated methods revealed several important insights into the effect of critical parameters on nanoparticle formation. Our study demonstrated that the incubation period of 10 days was optimal for AgNPs production. This suggests that the fungal metabolism and secretion of biomolecules required for reducing Ag^+^ ions and capping of nanoparticles are most active around this period. Shorter periods likely do not allow enough time for the fungi to produce sufficient quantities of these reducing and stabilizing agents. On the other hand, extending the incubation beyond 10 days did not significantly enhance the yield or properties of the nanoparticles, indicating that once the optimal conditions are met, further incubation does not improve nanoparticle formation. This aligns with previous studies highlighting the importance of incubation time in biological nanoparticle synthesis, where overly extended incubation periods can lead to by-product formation or degradation of nanoparticles [35, 36, 52].

The finding that 1 mM AgNO_3_ resulted in the most uniform and stable nanoparticles is in agreement with prior studies on nanoparticle synthesis. Lower concentrations, such as 0.25 and 0.5 mM, may not provide sufficient silver ions for effective reduction, limiting nanoparticle formation. Higher concentrations, however, tend to promote excessive nucleation, leading to particle aggregation and inconsistent size distribution. This suggests a delicate balance where sufficient silver ions must be present to drive the reduction process, but not so many that uncontrolled growth occurs, leading to large, polydisperse particles. These results highlight the importance of maintaining an optimal concentration of precursor to achieve controlled nucleation and growth of nanoparticles [34].

The pH of the reaction medium was shown to be a crucial factor in AgNPs synthesis. The optimal pH of 11 indicates that an alkaline environment enhances the reduction of silver ions by fungal metabolites. Alkaline conditions may increase the availability of functional groups in the fungal extract that act as reducing agents, leading to faster and more complete reduction of Ag^+^ to Ag^0^. These reducing agents may be protonated or less reactive at lower pH levels, resulting in incomplete nanoparticle formation. This finding is consistent with other research that has demonstrated the role of high pH in promoting efficient nanoparticle formation in biological synthesis methods [6, 40]. While synthesis at pH 11 maximizes yield, all biological testing used pH-neutralized AgNPs. The fungal protein corona’s stability prevents degradation, as confirmed by 3-month shelf-life studies (DLS data). This two-step process balances synthesis efficiency with biomedical applicability.

The influence of temperature was also significant, with higher temperatures producing superior results compared to lower temperatures. Higher temperatures increase the system’s kinetic energy, which accelerates the reduction of silver ions and the nucleation process, leading to faster and more efficient nanoparticle formation. Moreover, higher temperatures may enhance the activity of biomolecules involved in the reduction and stabilization of nanoparticles. However, excessively high temperatures may also lead to aggregation if the stabilizing agents are degraded or fail to cap the nanoparticles effectively. These observations underscore the necessity of maintaining a high but controlled temperature to optimize the synthesis of stable and well-dispersed AgNPs [38].

Beyond synthesis optimization, our findings reveal important mechanisms underlying AgNPs’ bioactivity. The antibacterial efficacy against Gram-negative pathogens (e.g., MIC of 2.5 µg/mL for *S*. *typhimurium*) likely stems from their small size (17.78 nm) and negative zeta potential (-16.5 mV), enabling electrostatic membrane adhesion and ROS-mediated damage [20, 64]. In MCF-7 cells, AgNPs induced apoptosis (Fig. 10) through cell cycle arrest and MMP-9 suppression, consistent with silver nanoparticles’ known interactions with cancer cell pathways [65, 66]. The fungal-derived protein (FT-IR peaks at 1635 cm⁻¹) appears to enhance this targeted bioactivity while maintaining stability, distinguishing our biogenic AgNPs from chemical alternatives [67].

AgNPs were applied in various crucial scientific fields, such as the creation of antibacterial agents [68], antifungal substances [69], anticancer treatments [70], antioxidant activities [71], and the catalytic degradation of Azo dyes [72].

Silver nanoparticles utilized as antibacterial agents in the current investigation. Microbial synthesized AgNPs have recently been applied in numerous medicinal uses like AgNPs fabricated by *A. terreus* were reported to prevent various harmful bacterial and fungal strains [34]. Also, AgNPs produced by *Aspergillus flavus* were used to avoid various pathogenic bacterial strains [40]. The proposed mechanism for metal nanoparticles’ antibacterial activity is that positively charged nanoparticles and negatively charged bacteria are attracted together by electrostatic attraction. The bacteria oxidized during this attraction and died instantly. Occasionally, the -SH groups on the surface proteins of bacteria interact with ions produced by nanoparticles to trigger cell death [64].

Breast carcinoma is a major reason of mortality for women worldwide, entailing the development of innovative treatment approaches. One promising avenue is using AgNPs derived from fungi or fungal extracts, due to their unique properties and potential anti-cancer effects. Flow cytometry analysis is a powerful tool that allows the examination of cellular characteristics and can provide valuable insights into the impacts of AgNPs on ‘MCF-7’ breast cancer cells. This study presents a flow cytometric analysis of control ‘MCF-7’ cells and those treated with AgNPs prepared using *A. templicola* filtrate, highlighting changes in cell cycle and apoptosis. Silver nanoparticles possess several characteristics that make them attractive candidates for cancer treatment. Their small size allows for easy penetration and accumulation within cancer cells. Moreover, their large surface area facilitates enhanced drug loading, bioavailability, and targeted delivery. Additionally, silver nanoparticles possess inherent antimicrobial properties, making them effective against infections that commonly arise during cancer therapy.

Studies have shown that AgNPs work against ‘MCF-7’ breast cancer cells through multiple mechanisms. First, by triggering intrinsic apoptotic pathways, these nanoparticles may initiate programmed cell death, or apoptosis, in cancer cells, and suppressing anti-apoptotic proteins. Second, silver nanoparticles can inhibit cellular proliferation by disrupting the mitotic process and inducing cell cycle arrest. Third, the development of new blood vessels that promote tumour growth, known as angiogenesis, can be inhibited by AgNPs. Lastly, silver nanoparticles have been observed to modulate key signaling pathways involved in cancer progression, such as the PI3K/Akt and MAPK pathways [65]. Also, Eco-friendly synthesized AgNPs may be used as a viable and affordable treatment option for this illness in the future. The ‘MCF-7’ cells proliferation was inhibited when treated with prepared AgNPs at IC_50_ (50 µg/mL) after 48 h. which may be due to the following treatment with AgNPs, which caused alterations in cell cycle distribution, such as an increase of cells number in the sub-G1 stage or cell cycle disruption in the G0 and G1 phases (apoptosis’s indicator) can be observed. Similarly, Ciftci et al. [73] reported that after 48 h of exposure, a rise in cell inhibition is seen, the ‘MCF-7’ cell lines’ apoptotic rate increases in response to an acceleration of AgNPs content in a dose-dependent way. According to their findings, ‘MCF-7’ cell lines were wholly inhibited (99%) at a maximal dose of 25 µg/mL. Also, at a 25 µL/mL concentration of AgNPs-fabricated ellagic acid was found to display 99% anti-proliferation [67].

The wound healing process involves a series of complex events, such as cell migration, proliferation, and extracellular matrix (ECM) remodeling. Silver nanoparticles possess unique properties, including antimicrobial activity and pro-regenerative effects, which make them suitable for accelerating wound closure for normal cells. The typical separation between the borders of each scratch is used to quantify the width of the wound. As cell migration increases, the wound width (mm) decreases. At current work Cisplatin helped cells to cure after 48 h while silver nanoparticles prevented the cancer cells from healing even at 72 h of treatment. According to Mariadoss et al. [74] who revealed that 33.81 µg/mL of *Malus domestica*-AgNPs dramatically reduced cancerous cell viability [75]. reported that using a mix of AgNPs and PDT groups ‘In the treatment of cancer, the application of a certain type of light via suitable photosensitizing agents, such as medications, nanoparticles, or chemicals, can decrease ‘MCF-7’ cell migration into the wound for a whole day [76]. Also, Parthasarathy et al. [77] recorded a cytotoxic effect of Chitosan-AgNPs for suppression of ‘MCF-7’ cell growth (64%) at 100 µg mL^− 1^. AgNPs may have this impact because they interact with cellular proteins during different wound repair stages of inflammation, proliferation, and maturation [78]. Also, this effect can be due to cytokine expression and migration of keratinocytes [79], while Sun et al. [66] said that AgNPs significantly impede the migration of cancer cells due to lowering expression of MMP-9; hence, the metastasis in human breast cancer is reduced.

While our green synthesis method demonstrates significant advantages in sustainability and bioactivity, three key limitations must be acknowledged: (1) The observed batch-to-batch size variation necessitates rigorous control of synthesis parameters (pH 11, 10-day incubation) to ensure reproducibility—a challenge common to biological nanoparticle synthesis. (2) Although the fungal protein enhances stability (PDI = 0.074) and biocompatibility (FT-IR), its complex composition may limit precise surface engineering for targeted drug delivery applications. Most critically, (3) while selective cytotoxicity to cancer cells was observed (5-fold lower MICs vs. IC50), comprehensive safety profiling in non-malignant human cell lines is underway to define therapeutic windows for clinical translation. These limitations represent the strategic foci of our current investigations into process optimization and functionalization protocols.

The expanding biomedical applications of AgNPs necessitate a thorough understanding of their potential organotoxicity. While AgNPs offer promising therapeutic benefits, their systemic distribution, whether through inhalation, ingestion, dermal contact, or injection—raises critical safety concerns. Therefore, the need for rigorous in vivo pharmacokinetic studies to optimize AgNP design—balancing therapeutic efficacy with biosafety—for future clinical translation must be addressed. In the current study, our system mitigates risks through: (1) fungal protein capping that prevents uncontrolled Ag⁺ release, (2) therapeutic doses 2.5–5× below cytotoxic thresholds, and (3) sterile production methods. Future studies should explore long-term exposure effects.

The expanding biomedical applications of AgNPs necessitate a thorough understanding of their potential organotoxicity. While AgNPs offer promising therapeutic benefits, their systemic distribution, whether through inhalation, ingestion, dermal contact, or injection—raises critical safety concerns. Studies indicate that administered AgNPs predominantly accumulate in metabolic and filtration organs (liver, spleen, kidneys, and lungs) [80, 81], with minimal deposition in mineralized tissues (bones, teeth). Of particular concern is the ability of smaller AgNPs (< 20 nm) to traverse biological barriers, including the blood-brain and blood-testis barriers, potentially leading to neurotoxicity or reproductive toxicity [82]. Moreover, nonspecific biodistribution may induce multi-organ toxicities, ranging from dermal irritation to hepatobiliary dysfunction, depending on exposure routes and nanoparticle characteristics (size, shape, concentration). These findings underscore the need for rigorous in vivo pharmacokinetic studies to optimize AgNP design—balancing therapeutic efficacy with biosafety—for future clinical translation.

In the current study, our system mitigates risks through: (1) fungal protein capping that prevents uncontrolled Ag⁺ release, (2) therapeutic doses 2.5–5× below cytotoxic thresholds, and (3) sterile production methods. Future studies should explore long-term exposure effects.

## Conclusions

This study demonstrates that *A*. *templicola* OR480102 filtrate provides a highly efficient, eco-friendly platform for synthesizing stable, spherical AgNPs (5–25 nm) with dual therapeutic functionality, exhibiting potent antibacterial activity against clinically relevant pathogens (MICs 2.5–20 µg/mL) for potential medical textile applications, and selective anticancer effects through sustained inhibition of MCF-7 migration (72 h) and apoptosis induction, highlighting promise contributions for oncology. The fungal protein corona (FT-IR-confirmed) ensures long-term stability, addressing a critical translational challenge. Future work will optimize therapeutic ratios by probing AgNP-protein interactions, develop targeted delivery systems (e.g., hydrogels or functionalized textiles), and validate biosafety in non-malignant cells and in vivo models to bridge these findings toward clinically relevant therapeutic strategies.

## Data Availability

All data generated or analyzed during this study are included in this published article. The obtained 18S rRNA nucleotide sequence of A. templicola MAK 223 is available from NCBI under accession number OR480102.
